
JOURNAL INFORMATION
==============================
NLM Title Abbreviation: Biospektrum (Heidelb)
Journal ID: Biospektrum (Heidelb)
NLM Journal ID: 9615685

Biospektrum

ISSN: 0947-0867
EISSN: 1868-6249

ARTICLE INFORMATION
==============================
PMCID: PMC8417631
PMID: 34511735
DOI: 10.1007/s12268-021-1623-3
Article ID: 1623
Article version: 1
Subjects: Wissenschaft Special

Vom Huhn abgeleitete Antikörper für Diagnostik und Immuntherapie

Elter Adrian 1 2
Bogen Jan P. 1 3
Habermann Jan 1
Kolmar Harald Kolmar@Biochemie-TUD.de
1 4
1 grid.6546.1 0000 0001 0940 1669 Institut für Organische Chemie und Biochemie, TU Darmstadt, Darmstadt, Deutschland
2 grid.6546.1 0000 0001 0940 1669 Merck Lab @ TU Darmstadt, Darmstadt, Deutschland
3 Ferring Darmstadt Laboratory, Darmstadt, Deutschland
4 grid.6546.1 0000 0001 0940 1669 Angewandte Biochemie, Technische Universität Darmstadt, Alarich-Weiss-Straße 4, D-64287 Darmstadt, Deutschland
Electronic publication date: 2021 Sep 4
Print publication date: 2021
Volume: 27
Issue: 5
First page: 500
Last page: 504
Copyright: © Die Autoren 2021
License: Dieser Artikel wird unter der Creative Commons Namensnennung 4.0 International Lizenz veröffentlicht, welche die Nutzung, Vervielfältigung, Bearbeitung, Verbreitung und Wiedergabe in jeglichem Medium und Format erlaubt, sofern Sie den/die ursprünglichen Autor(en) und die Quelle ordnungsgemäß nennen, einen Link zur Creative Commons Lizenz beifügen und angeben, ob Änderungen vorgenommen wurden. Die in diesem Artikel enthaltenen Bilder und sonstiges Drittmaterial unterliegen ebenfalls der genannten Creative Commons Lizenz, sofern sich aus der Abbildungslegende nichts anderes ergibt. Sofern das betreffende Material nicht unter der genannten Creative Commons Lizenz steht und die betreffende Handlung nicht nach gesetzlichen Vorschriften erlaubt ist, ist für die oben aufgeführten Weiterverwendungen des Materials die Einwilligung des jeweiligen Rechteinhabers einzuholen. Weitere Details zur Lizenz entnehmen Sie bitte der Lizenzinformation auf http://creativecommons.org/licenses/by/4.0/deed.de.
License URL: https://creativecommons.org/licenses/by/4.0/

issue-copyright-statement: © Springer-Verlag GmbH Deutschland, ein Teil von Springer Nature 2021

==============================
Due to the large evolutionary distance between birds (Aves) und humans, immunization of chickens with human proteins results in a strong response of the bird’s adaptive immune system to proteins of mammalian origin. Additionally, chicken-derived antibodies display less undesired cross-reactivity in analytical setups than conventional rodent-derived antibodies. Due to these features as well as the facile amplification of antibody-coding genes, chicken-derived antibodies emerged as promising molecules for the immunotherapy and various biotechnological applications.

◀ **Abb. 1:** Unterschiede zwischen humanen und murinen IgG sowie Hühnerantikörpern. Phylogenetischer Stammbaum mit evolutionärer Distanz zwischen Huhn, Maus und Mensch. Die Antikörper-Fc-Teile sind als Kristallstruktur dargestellt (PDB-IDs: humaner IgG 6KRU, muriner IgG 5JII, IgY 2W59). Der humane IgG1-Fc ist in blau dargestellt. Abweichungen in den Aminosäuren im murinen Fc und Hühner-Fc sind rot markiert. Erstellt mit BioRender.com.

■ In den letzten Monaten wird die Gewinnung von Antikörpern durch die Immunisierung von Versuchstieren sehr kontrovers diskutiert. Einige Forschende plädieren für die tierfreie Gewinnung monoklonaler Antikörper ausschließlich aus Masterbibliotheken, die aus dem Blut nicht immunisierter Spender gewonnen und mit speziellen molekularbiologischen Methoden wie Phagen-Display nach Antikörpern mit gewünschten Eigenschaften durchmustert werden [1]. Solche Ansätze erfordern den Einsatz sehr großer Bibliotheken mit mehreren Milliarden Varianten. Es braucht viel Erfahrung in der Handhabung und der Isolierung von Klonvarianten mit gewünschten Bindeeigenschaften. Diese Verfahren bleiben daher auf wenige spezialisierte Labore oder Biotechunternehmen, die die Antikörperisolierung als bezahlten Service anbieten, beschränkt [2]. Der klassische Ansatz zur Antikörpergewinnung geht von der Immunisierung von Versuchstieren aus. Auch hier verfügen Labors mittlerweile über eine Vielzahl von Methoden, um maßgeschneiderte monoklonale Antikörper zu gewinnen. Der große Vorteil des Immunisierungsansatzes liegt darin, dass der natürliche Reifungsapparat zur Generierung von Antikörpern genutzt wird und dadurch aus kleineren Bibliotheken hochaffine und hochspezifische Antikörper erhalten werden können, die bereits einer natürlichen Funktions- und Stabilitätsselektion durch die B-Zellreifung unterlagen. Wir haben experimentelle Erfahrung mit beiden Strategien gemacht und in den letzten Jahren festgestellt, dass sich insbesondere immunisierte Hühner in hervorragender Weise für die Gewinnung monoklonaler Antikörper eignen.

### IgY – strukturelle Merkmale

Das adaptive Immunsystem des Haushuhns (*Gallus gallus domesticus*) besitzt neben anderen Vögeln, sowie einigen Amphibien und Reptilien einen besonderen Antikörperisotyp. Die Immunoglobuline Y (IgY), welche aus dem Hühnereigelb (engl. Yolk) in großen

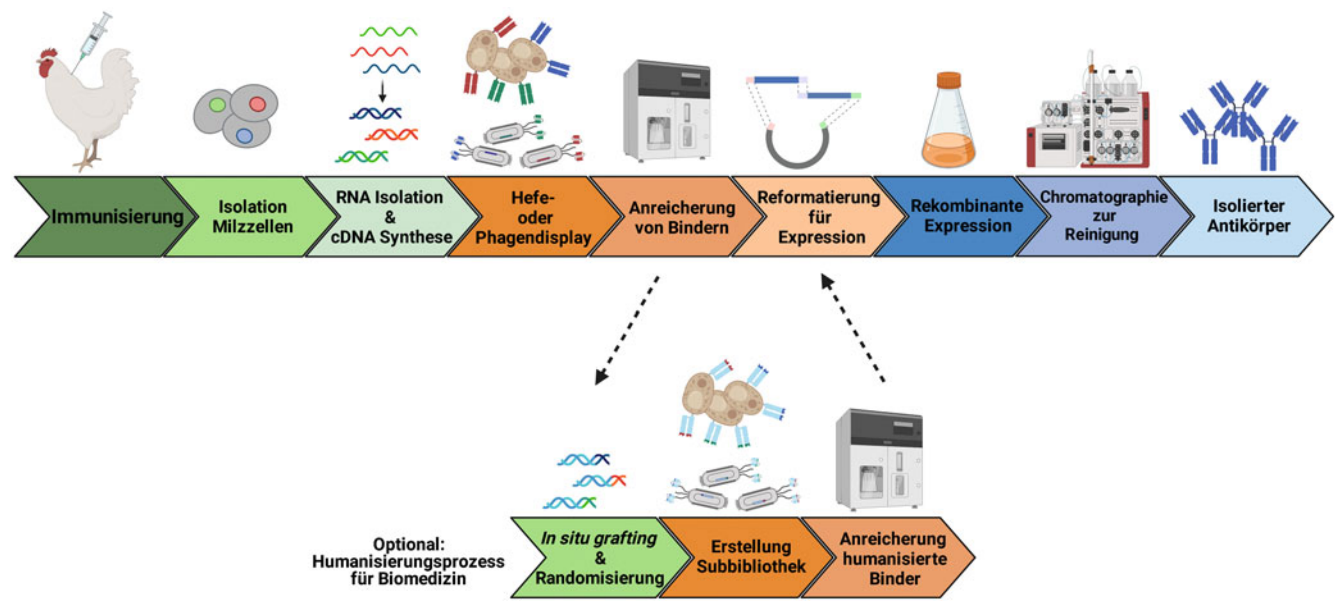

▲ **Abb. 2:** Vom immunisierten Huhn zum isolierten Antikörper. Nach der Immunisierung und der Isolierung der Milz können mit molekularbiologischen Methoden Immunbibliotheken in verschieden Display-Formaten aufgebaut werden. Durch Selektionsmethoden wie FACS werden Antigenbinder isoliert. Optional kann nach der Anreicherung von Bindern ein Humanisierungsprozess durchgeführt werden. Hierzu wird per *in situ graftig* der CDRs und mittels Teilrandomisierung von Proteinregionen eine Subbibliothek erstellt. Anschließend können per Selektion humanisierte Binder isoliert werden. Nach rekombinanter Expression werden die Binder mittels chromatographischer Reinigung aufgearbeitet und funktionell charakterisiert. Erstellt mit BioRender.com.

Hier steht
eine Anzeige.

Springer

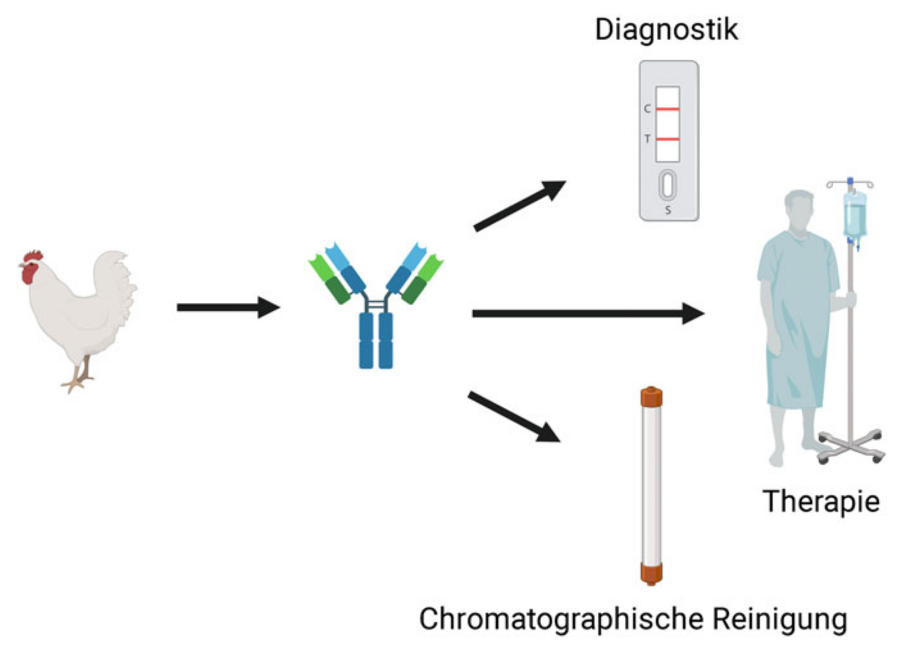

▲ **Abb. 3:** Anwendungsgebiete von Huhnantikörpern: Biomedizin, Diagnostik und Biotechnologie. Erstellt mit BioRender.com.

Mengen gewonnen werden können, wurden erstmals 1969 von A. Leslie und L.W. Clem detailliert charakterisiert [3].

IgYs bestehen wie Säugetierantiköper aus zwei schweren und zwei leichten Ketten (**Abb. 1**). Wie bei Säugetierantikörpern findet man in den variablen Bindedomänen der IgY je drei Bindeschleifen (*complementarity determinding regions*), CDR1, CDR2 und CDR3 (**Abb. 1**). Bei großer struktureller Ähnlichkeit zu humanen IgG-Antikörpern findet sich jedoch eine geringe Übereinstimmung in den Aminosäuresequenzen.

## Generierung von monoklonalen Huhnantikörpern

Durch die phylogenetische Distanz zwischen Menschen und Vögeln und der einhergehenden niedrigen proteomischen Homologie identifiziert das Immunsystem eines Huhns in der Regel den Großteil eines zur Immunisierung applizierten humanen Proteins als körperfremd. Daraufhin wird das adaptive Immunsystem des Huhns stimuliert, in dessen Folge spezifische Antikörper generiert werden, welche Regionen (Epitope) des Antigens erkennen und binden. In Säugetieren hingegen ist die Immunantwort bei einer Vakzinierung mit einem humanen Antigen aufgrund seiner Sequenz- und Strukturhomologie zu eigenen Proteinen oft nur gering und man beobachtet nicht selten eine geringe Diversität der erhaltenen monoklonalen Antikörper und daraus resultierend eine geringe Epitopabdeckung [4]. Üblicherweise werden monoklonale Antiköper (mAbs) und Antikörperfragmente durch Einsatz verschieder Verfahren aus immunisierten Tieren isoliert. Neben dem klassischen Hybridoma-Ansatz [5] haben sich Immunbibliotheken in Kombination mit verschieden Display-Verfahren bewährt. Neben dem Phagen-Display als Methode der Wahl zur Durchmusterung von Huhn-Immunbibliotheken nach spezifischen Bindern [6] eignet sich auch Hefe-Display perfekt für die Isolierung von Bindern gegen diverse Antigene durch fluoreszenzaktivierte Hochdurchsatzzellsortierung (FACS). Dabei können nicht nur lösliche, sondern mittlerweile auch membranständige Targets adressiert werden. Unsere Arbeitsgruppe hatte erst kürzlich in einem neuartigen Verfahren eine Huhn-Immunbibliothek im Hefe-Display im Hochdurchsatz gegen Antigene auf der Oberfläche von Tumorzellen erfolgreich durchmustert [7]. Neben Phagen- und Hefe-Display sind außerdem in der Literatur Verfahren beschrieben, Antikörper aus Hühner-Immunbibliotheken auf der Oberfläche von Säugerzellen zur Durchmusterung bereitzustellen (Mammalian-Display [8]) oder direkt bestimmte Immunzellen des Huhns zu isolieren, welche Antikörper gegen das gewünschte Antigen produzieren und danach die codierende Antikörpersequenzen zu isolieren [9].

Als sehr hilfreich für die Isolierung von Antikörpergenen hat sich erwiesen, dass die Anfangs- und Endbereiche der Antikörpercodierenden Genregionen der Hühner stark konserviert sind. Dieser Umstand ermöglicht die einfache und schnelle Extraktion und Amplifizierung der genetischen Informationen der gesamten Antikörperdiversität mittels PCR.

Ein exemplarisches Verfahren zur Generierung von antigenspezifischen Antikörperfragmenten aus einem immunisierten Huhn durch Hefe-Display und FACS ist in **Abbildung 2** dargestellt.

## Huhnantikörper für die biotechnologische und diagnostische Anwendung

IgYs interagieren nicht mit humanen Serumbestandteilen (Komplementfaktoren) oder Fc-Rezeptoren auf Blutzellen und eignen sich damit sehr gut für die Analytik von Patientenproben [10]. Unsere Arbeitsgruppe konnte zeigen, dass Antikörperfragmente aus Hühnern in Schnelltests (*lateral flow tests*) die sensitive Detektion des Schwangerschaftshormons hCG ermöglichen [11]. In einer weiteren Studie wurde die Anwendung eines Hühnerantikörpers in einem papierbasierten COVID-19-Antikörperschnelltest demonstriert [12]. Ebenso konnte der Nutzen von Hühnerantikörpern für die Detektion von humanen Autoantigenen [13] und Parasitenantigenen gezeigt werden [14]. Ein weiteres Anwendungsbeispiel ist die chromatographische Reinigung von humanen Antikörpern. Mithilfe der Hefe-Display-Technologie konnten pH- und Kation-responsive Huhnantikörper identifiziert werden, welche, immobilisiert als Affinitätsliganden, für die schonende und kostengünstige chromatographische Proteinaufreinigung eingesetzt werden können [15].

## Huhnantikörper für die Biomedizin

Um Antikörper aus Säugetieren und auch aus Hühnern in der Biomedizin einsetzen zu können, ist eine Antikörperhumanisierung notwendig. Das menschliche Immunsystem identifiziert Huhnantikörper als fremd, was eine Immunreaktion gegen das therapeutische Protein induzieren würde. Neben einer verkürzten Halbwertszeit wird dadurch oft auch die Wirksamkeit des therapeutischen mAbs stark eingeschränkt, bis hin zum vollständigen Wirksamkeitsverlust [16]. Durch die strukturelle Ähnlichkeit der variablen Domänen der Huhnantikörper und menschlicher Antikörper lassen sich die CDRs, welche für die Antigenbindung verantwortlich sind, mittels *in situ grafting* und Teilrandomisierung von einzelnen Proteinbausteinen auf humane Antikörperstrukturen übertragen [17]. Dies resultiert in humanisierten Huhnantikörpern mit potenziell verringerter Immunogenität im Menschen.

Der erste humanisierte Huhnantikörper, welcher in klinischen Phasen getestet wurde, war der anti-PD1-Antikörper Sym021 von Symphogen A/S. Dieser blockiert die Interaktion von PD-1 auf T-Zellen mit PD-L1 auf Tumorzellen und fungiert so als Checkpointinhibitor [18]. Derzeit laufen mehrere klinische Phase-I-Studien mit Sym021 als Mono- oder Kombinationstherapie in Patienten mit vorangeschrittenen Krebserkrankungen (NCT03311412, NCT04641871, NCT04672434). Der Übergang von Sym021 von der Präklinik in frühe klinische Phasen unterstreicht das Potenzial von Huhnantikörpern für therapeutische Anwendungen am Menschen.

In den letzten Jahren gelangten insbesondere auch bispezifische Antikörper in den Fokus der biopharmazeutischen Forschung. Diese Antikörper bestehen aus zwei verschiedenen schwere und leichte Kettenpaaren, welche zwei unterschiedliche Antigene (bispezifisch) oder zwei unterschiedliche Epitope auf einem Antigen (biparatopisch) erkennen können und synergistische therapeutische Wirkungen vermitteln. Unsere Arbeitsgruppe etablierte, basierend auf immunisierten Hühnern, eine Plattform zur Generierung von biparatopischen Antikörpern, die durch die Verwendung einer einzelnen leichten Kette für beide Fab-Arme die Gefahr einer falschen Kettenpaarung umgeht. Der biparatopische anti-EGFR-Antikörper verfügt über eine verbesserte Affinität und eine gesteigerte antikörperabhängige zellvermittelte Cytotoxizität (ADCC) gegen Krebszellen [19]. Basierend auf diesen Ergebnissen wurde ein trispezifischer humanisierter Huhnantikörper entwickelt, in welchem drei Fab-Arme die identische leichte Kette akzeptierten. Während zwei Fab-Arme die Tumorzellen adressieren und sowohl als Checkpoint- als auch als Wachstumsinhibitor fungieren, rekrutiert der dritte Fab-Arm cytotoxische Immunzellen [20].

## Fazit

Moderne molekularbiologische Methoden liefern in Kombination mit Tierimmunisierung sehr verlässlich mit vergleichsweise geringem experimentellem Aufwand funktionell gut charakterisierte Antikörper für den diagnostischen und therapeutischen Einsatz [21]. Die vollständige Abschaffung von Tierimmunisierungen, die das EU-Referenzlabor für Alternativen zu Tierversuchen EURL EVCAM fordert, halten wir nach derzeitigem Wissenschaftsstand für unverantwortlich. Dies würde u. a. die Entwicklung therapeutischer Antikörper für die Behandlung einer Vielzahl von Erkrankungen massiv beeinträchtigen. Allerdings sollte hoffentlich bald die Zeit vorbei sein, in der schlecht charakterisierte Antikörper mit unzureichenden Bindungseigenschaften in den Umlauf kommen. Moderne Methoden der Antikörperisolierung und ausgefeilte Analytikverfahren liefern mittlerweile ein verlässliches technisches Rüstzeug für die Isolierung und ausführliche funktionelle Validierung dieser nach wie vor für die Biologie und Medizin unverzichtbaren Moleküle. ■

Hier steht
eine Anzeige.

Springer

## ARBEITSGRUPPE

Hier steht
eine Anzeige.
Springer


Funding note

Open Access funding enabled and organized by Projekt DEAL.

Die Arbeitsgruppe Angewandte Biochemie um Prof. Dr. Harald Kolmar (unten rechts) ist seit 2005 am Fachbereich Chemie der TU Darmstadt angesiedelt. Die Doktoranden Adrian Elter (oben links), Jan P. Bogen (oben rechts) und Jan Habermann (unten links) forschen zusammen an verschiedenen Aspekten des Antikörper-Engineering. Die Forschung zielt auf die Generierung und funktionelle Charakterisierung von Proteinen und Peptiden mit therapeutischer Relevanz ab. Ein Fokus liegt dabei auf dem Gebiet des Antikörper-Engineering und der Erzeugung maßgeschneiderter multifunktioneller Antikörper für die Tumortherapie.


Literatur

[1] Gray A Bradbury ARM Knappik A Animal-free alternatives and the antibody iceberg Nat Biotechnol 2020 38 1234 1239 10.1038/s41587-020-0687-9 33046876
[2] Custers R Steyaert J Discussions on the quality of antibodies are no reason to ban animal immunization EMBO Rep 2020 21 e51761 10.15252/embr.202051761 33179844 PMC7726777
[3] Leslie GA Clem LW Phylogen of immunoglobulin structure and function. 3. Immunoglobulins of the chicken J Exp Med 1969 130 1337 1352 10.1084/jem.130.6.1337 5352783 PMC2138693
[4] Ching KH Collarini EJ Abdiche YN Chickens with humanized immunoglobulin genes generate antibodies with high affinity and broad epitope coverage to conserved targets MAbs 2018 10 71 80 10.1080/19420862.2017.1386825 29035625 PMC5800366
[5] Nishinaka S Matsuda H Murata M Establishment of a chicken X chicken hybridoma secreting specific antibody Int Arch Allergy Appl Immunol 1989 89 416 419 10.1159/000234985 2793228
[6] Andris-Widhopf J Rader C Steinberger P Methods for the generation of chicken monoclonal antibody fragments by phage display J Immunol Methods 2000 242 159 181 10.1016/S0022-1759(00)00221-0 10986398
[7] Bogen JP Sorka J Yanakieva D Isolation of common light chain antibodies from immunized chickens using yeast biopanning and fluorescence-activated cell sorting Biotechnol J 2021 16 e2000240 10.1002/biot.202000240 32914549
[8] Akamatsu Y Pakabunto K Xu Z Whole IgG surface display on mammalian cells: Application to isolation of neutralizing chicken monoclonal anti-IL-12 antibodies J Immunol Methods 2007 327 40 52 10.1016/j.jim.2007.07.007 17719061
[9] Mettler Izquierdo S Varela S Park M High-efficiency antibody discovery achieved with multiplexed microscopy Microscopy (Oxf) 2016 65 341 352 10.1093/jmicro/dfw014 27107009 PMC5895110
[10] Larsson A Bålöw RM Lindahl TL Forsberg PO Chicken antibodies: taking advantage of evolution — a review Poult Sci 1993 72 1807 1812 10.3382/ps.0721807 8415358
[11] Grzeschik J Yanakieva D Roth L Yeast surface display in combination with fluorescence activated cell sorting enables the rapid isolation of antibody fragments derived from immunized chickens Biotechnology J 2019 14 e1800466 10.1002/biot.201800466 30350923
[12] Elter A Bock T Spiehl D Carbohydrate binding module-fused antibodies improve the performance of cellulose-based lateral flow immunoassays Sci Rep 2021 11 7880 10.1038/s41598-021-87072-7 33846482 PMC8042022
[13] Hof D Hoeke MO Raats JM Multiple-antigen immunization of chickens facilitates the generation of recombinant antibodies to autoantigens Clin Exp Immunol 2008 151 367 377 10.1111/j.1365-2249.2007.03569.x 18062792 PMC2276936
[14] Thirumalai D Ambi SV Vieira-Pires RS Chicken egg yolk antibody (IgY) as diagnostics and therapeutics in parasitic infections — a review Int J Biol Macromol 2019 136 755 763 10.1016/j.ijbiomac.2019.06.118 31220492
[15] Hinz SC Elter A Rammo O A generic procedure for the Isolation of pH- and magnesium-responsive chicken scFvs for downstream purification of human antibodies Front Bioeng Biotechnol 2020 8 688 10.3389/fbioe.2020.00688 32656201 PMC7324474
[16] Ulitzka M Carrara S Grzeschik J Engineering therapeutic antibodies for patient safety: tackling the immunogenicity problem Protein Eng Des Sel 2020 33 gzaa025 10.1093/protein/gzaa025 33128053
[17] Elter A Bogen JP Hinz SC Humanization of chicken-derived scFv using yeast surface display and NGS data mining Biotechnol J 2021 16 e2000231 10.1002/biot.202000231 33078896
[18] Gjetting T Gad M Fröhlich C Sym021, a promising anti-PD1 clinical candidate antibody derived from a new chicken antibody discovery platform MAbs 2019 11 666 680 10.1080/19420862.2019.1596514 31046547 PMC6601539
[19] Bogen JP Carrara SC Fiebig D Expeditious generation of biparatopic common light chain antibodies via chicken immunization and yeast display screening Front Immunol 2020 11 606878 10.3389/fimmu.2020.606878 33424853 PMC7786285
[20] Bogen JP Carrara SC Fiebig D Design of a trispecific checkpoint inhibitor and natural killer cell engager based on a 2+1 common light chain antibody architecture Front Immunol 2021 12 669496 10.3389/fimmu.2021.669496 34040611 PMC8141644
[21] Bogen JP Grzeschik J Krah S Rapid generation of chicken immune libraries for yeast surface display Methods Mol Biol 2020 2070 289 302 10.1007/978-1-4939-9853-1_16 31625102
